# Using *Hermetia illucens* to process Ugandan waragi waste

**DOI:** 10.1016/j.jclepro.2018.11.176

**Published:** 2019-02-20

**Authors:** Darja Dobermann, Lin M. Field, Louise V. Michaelson

**Affiliations:** a Rothamsted Research, Harpenden, United Kingdom; b Department of Nutritional Sciences, University of Nottingham, Nottingham, United Kingdom

**Keywords:** Hermetia illucens, Waste management, Waragi, Waste reduction, Sustainable feed

## Abstract

Waragi, a form of homemade gin, is produced throughout Uganda in both legal and illegal breweries. Waste produced during the illegal brewing process is predominantly disposed of via indiscriminate dumping into surrounding environments and reports from local communities have indicated this to be harmful to crops and livestock. The larvae of *Hermetia illucens* are documented to consume a wide range of otherwise unappealing waste products. In addition to this, the prepupal stages of the larvae can serve as a high-quality protein feed for animal livestock. Therefore, the feasibility of the larvae of *H. illucens* to digest waragi waste was evaluated. A dietary toxicity trial was run to establish an LC_50_ value for waragi inclusion in larval diets. This was followed by a larger scale trial utilising waragi waste in combination with various in situ available feed stuffs to further assess the viability of processing waragi waste using *H. illucens.* Larvae were able to eat diets composed of up to 85% waragi waste without any significant impact (*p* > .01) on survival or growth. When combined with locally available feed sources, e.g. chicken offal, cottonseed cake, sunflower meal or groundnut cake, larvae showed high survival (>95%) and growth rates on diets including 25% waragi waste. Results indicate that *H. illucens* larvae may be a useful tool in processing waragi waste.

## Highlights

• Hermetia illucens larvae are examined as a possible mechanism to valorise waragi waste.
• Toxicity trials show no significant impact on growth or survival up to 85% dietary waragi waste inclusion.
• 25% waragi waste combined with additional in situ waste products demonstrated high survival and growth rates of larvae.
• Diets containing waragi waste had no impact on subsequent development and reproduction of H. illucens.

## Graphical abstract

**Figure:**
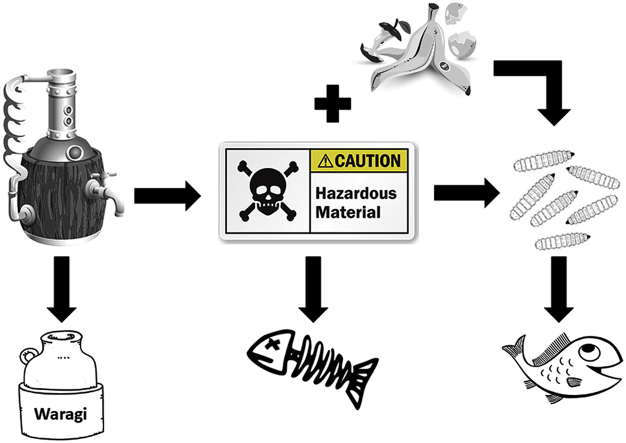

## 1 Introduction

Adequately managing hazardous waste by-products is vital to protecting public health and preventing environmental damage. This is particularly true in Sub-Saharan Africa where 28% of the population relies on water from unprotected wells or surface water (e.g. rivers, lakes, canals) (UNICEF and WHO, 2017). With nearly 10% of the global burden of disease attributed to lack of access to clean water ensuring that water stays as safe as possible is paramount (Prüss-Üstün et al., 2008). Additionally, water for agricultural land, if its irrigated, is drawn or diverted from rivers and lakes resulting in an increased risk of crop damage from contaminated water.

One such contaminant risk is the unregulated dumping of waste by-products from the illegal waragi home-brewing industry in Uganda (Republic of Uganda, n.d.). Waragi is a local gin spirit brewed from molasses and cassava/millet flour and its unregulated production accounts for 80% of the liquor produced in Uganda (Gatsiounis, 2010; Tumwine and Munguti, 2002). There is no research published on the impact of waragi waste dumping on the environment in Uganda or on why it is hazardous. However, there is ample anecdotal evidence from local newspapers and radio shows to support that the dumping has significantly polluted drinking water, killed fish, damaged agricultural land and crops, and caused health issues in individuals who have come in contact with contaminated water (Elunya, 2009; Kasamba and Mugo, 2007; Ssebuyira, 2014; Ssekika, 2015).

One prospective solution for processing the waragi waste is the larvae of *Hermetia illucens*, known commonly as the black soldier fly. *H. illucens* larvae are detrivores and significant research has demonstrated that the larvae are capable of processing livestock manure, human faecal waste, and food waste without difficulty (Banks et al., 2014; Newton et al., 2005; Oonincx et al., 2015a, Oonincx et al., 2015b; Sheppard et al., 1994). In fact, research demonstrated the larvae were able to reduce *the Escherichia coli* and *Salmonella enterica servor enteriditis* load within livestock manure and human faecal waste, making it safer to subsequently dispose of (Erickson et al., 2004; Lalander et al., 2013; Liu et al., 2008). After 10 days, the larvae also showed no further traces of *E. coli* or *Salmonella* within their own digestive systems, making them safe to utilise further.

*H. illucens* larvae are predominantly utilised as an animal feed stuff due to their valuable nutritional profile. On average, larvae are reported as consisting of 40% protein and 35% fat (Newton et al., 2005; Sheppard et al., 1994), though these values can range from 37 to 67% protein and 7–39% fat depending on the feeding substrate of the larvae (Barragan-Fonseca et al., 2017; Tschirner and Simon, 2015). They also have an amino acid profile closely resembling fish meal (Tschirner and Simon, 2015; Elwert et al., 2010) making them an ideal alternative to expensive fish meal in omnivorous fish farming. Studies feeding *H. illucens* larvae as part of other feed stuffs to chicken, pigs, catfish and tilapia have demonstrated predominantly positive results (Barragan-Fonseca et al., 2017; Sealey et al., 2011; Elwert et al., 2010; Bondari and Sheppard, 1987; Hale, 1973). The cost of livestock feed, both economically and environmentally, is exerting increasing pressure on food systems, particularly for small-holder farmers (McDermott et al., 2010). Finding sustainable, affordable alternatives is paramount and *H. illucens* is one option.

Lastly, the adult flies are not a nuisance species or a mechanical disease vector. Owing to the fact that the adult flies do not feed, living off fat stores, and female flies only ovipost on the edges of larval food sources avoiding contact with harmful pathogens (Copello, 1926; Furman et al., 1959). The presence of *H. illucens* larvae in waste also serves to deter oviposition of other species such as *Musca domestica* which do act as a vector to spread disease (Furman et al., 1959; Sheppard, 1983).

These factors combined make *H. illucens* larvae the ideal candidate species for potentially processing harmful waragi brewing waste and preventing it from contaminating the environment. This trial examines the viability of this solution by examining at which, if any, level of inclusion in the diet waragi waste becomes toxic to *H. illucens* larvae and testing the impact of experimental diets which include waragi waste and other feed ingredients locally available in Uganda on the growth, survival and development of larvae.

## 2 Materials and methods

### 2.1 Feed sources

The waragi brewing waste was sourced directly from a waragi distiller in the Mbale district, Uganda. Due to the illegal nature of waragi brewing its production is not strictly standardised. However, it is generally produced using a combination of molasses, pure ethanol and other unidentified chemicals which are fermented in old oil drums and then distilled resulting in a black, sticky waste by-product (Heath, 2015).

Organic millet flakes were purchased from Tree of Life UK Ltd., product is sourced from various countries and packaged in the UK. Groundnut cake, sunflower seed meal and cotton seed cake were sourced from local feed producers in Uganda. Chicken offal was sourced from a butcher in the United Kingdom. If necessary, feed was ground to attain homogeneity. All feeds were made fresh every 2–3 days and stored at 4 °C.

### 2.2 Dietary toxicity trial

A dietary toxicity trial was set-up to initially establish at which level, if any, of inclusion in the diet waragi waste becomes harmful to *H. illucens* larvae. This trial was modelled after standard Organisation for Economic Co-Operation and Development Guidelines for Testing of Chemicals in both insects and poultry (OECD, 2012, 1995, 1984). Table 1 displays the nine dosage levels and the feed composition used for the larvae. Chicken mash (Fancy Feed Layers' Mash) was used as the base feed for all diets and all diets were formulated to a minimum of 60% moisture content (Cheng et al., 2017; Diener et al., 2009). Waragi waste was used in its natural liquid form as methods for drying it would not be available in situ, due to this any dosage above 65% waragi had a moisture content also above 65%

**Table 1:** Dietary formulation of dosage levels in dietary toxicity trial.

	% Waragi	% Chicken mash	% Water	% Moisture
Dosage level				
Control	0	35	65	65
D1	25	35	40	65
D2	35	35	30	65
D3	45	35	20	65
D4	55	35	10	65
D5	65	35	0	65
D6	75	25	0	75
D7	85	15	0	85
D8	95	5	0	95

Diets were made up fresh every two days and stored at 4 °C in between feeding. Food was available *ad libitum* to the larvae and larvae were checked twice a day to ensure food was available.

Each dosage level contained 5 replicates with 30 larvae per replicate. Seven-day old larvae of *H. illucens* were obtained from Entomics Biosystems Ltd., they were previously fed on a starter diet comprised of vitamins, egg yolk, protein powder, bran and semolina (exact recipe is withheld to protect company). Larvae were placed in plastic rearing pots with mesh lids, no other substrate was provided; pots were kept in a climate control chamber at 30 °C with 70–75% humidity and 14-h days.

Survival was measured daily for each replicate for 72 h and then again at the first signs of pupation, indicated by a change in colour from light to dark (Tomberlin et al., 2009). Individual larvae were also weighed daily for 72 h to monitor growth and again at the first signs of pupation, larvae were not washed prior to weighing as previous research has indicated it does not significantly impact weight (Banks et al., 2014). Lethal concentration (LC_50_) was defined as the percent inclusion of waragi which resulted in 50% mortality of the larvae.

### 2.3 Pilot feeding trial

A pilot trial was conducted to identify which locally available feed ingredients in Uganda could be used in combination with the waragi waste to ensure the end diet was nutritionally suitable for growth in situ. Initial diets were composed predominantly of millet flakes, however impaired larval growth (significantly smaller larvae, *p* < .05) was observed over a two-week period compared to the groups on chicken feed. Given the nutritional composition of millet flakes at approximately 75% carbohydrates (USDA, n.d.) it was concluded this growth restriction was likely due to low protein levels in the feed, which has been previously demonstrated to significantly impact growth (Oonincx et al., 2015a, Oonincx et al., 2015b). As such four feed sources (chicken offal, groundnut cake, sunflower seed meal, and cottonseed cake) higher in protein were identified and incorporated into the diets.

### 2.4 Feeding trial

The feeding trial was designed to examine the impact of including waragi waste in the diets of *H. illucens* larvae on growth, survival and development. Four experimental and one control diet were examined in triplicate. The amount of waragi waste in the experimental diets was held constant at 25%, as this was an amount which would be reasonably available in situ and was well in line with results from the dietary toxicity portion of the study.

All diets were formulated to a protein level of 15% (±0.04) with a moisture content of 60%. All ingredients were dry excluding the chicken offal, moisture content of the offal was established and accounted for in composition calculations. The main variation between diets was the primary source of protein. The protein came from either chicken mash (Control), chicken offal (CP), groundnut cake (GN), sunflower seed meal (SSM) or cottonseed cake (CSC). Table 2 lists detailed diet compositions.

**Table 2:** Diet composition, by percent inclusion of ingredients.

	Control	CP	GN	SSM	CSC
Ingredient					
Chicken Mash	36	–	–	–	–
Wheat Bran	4	–	–	–	–
Millet Flakes	–	36	26.2	18.4	31.2
Chicken Offal	–	15.2	–	–	–
Groundnut Cake	–	–	13.8	–	–
Sunflower Seed Meal	–	–	–	21.6	–
Cottonseed Cake	–	–	–	–	8.8
Waragi	–	25	25	25	25
Water	60	24	35	35	35

Seven-day old larvae of *H. illucens* were obtained from Entomics Biosystems Ltd again. 300 larvae were placed in plastic containers with a volume of 650 ml (17 cm L x 11.5 cm W x 5.5 cm D) of which the bottom had been replaced with geotextile material. Each container contained 1 cm of coconut coir in the bottom to provide larvae with enough substrate to burrow in and maintain adequate moisture levels; previous trials demonstrated the larvae would not consume coconut coir. Larvae containers were housed at 30 °C with 14-h day conditions, and 60% humidity, substrate was misted with water throughout trial as needed to maintain moisture levels.

Fresh feed was provided daily to larvae to avoid fungal growth in the cages, sufficient feed was provided to ensure *ad libitum* feeding. A subset of 15 individuals was weighed from each replicate every other day to monitor growth. Feeding and weighing continued until first signs of pupation.

At pupation, larvae numbers for each replicate were counted to assess survival. Pupae were placed in moist clean coconut coir and returned to climate chamber to incubate. Adult *H. illucens* from each group were counted to establish emergence rates and ensure diet did not negatively impact development; previous work has demonstrated sub-optimal diets to impact adult development (Gobbi et al., 2013). Development time was defined as the time from larval eclosion to the signs of the first prepupae.

### 2.5 Statistical analysis

Data were analysed using IBM SPSS Statistics 22 (IBM Corporation, New York) and Microsoft Excel (Microsoft, Washington) to look for differences between groups using one-way between subjects analysis of variance (ANOVA). Average daily weight gain was calculated by taking the starting weight of individual larvae minus the end weight divided by the length of the trial (in days).

## 3 Results and discussion

### 3.1 Dietary toxicity

**Fig. 1:**
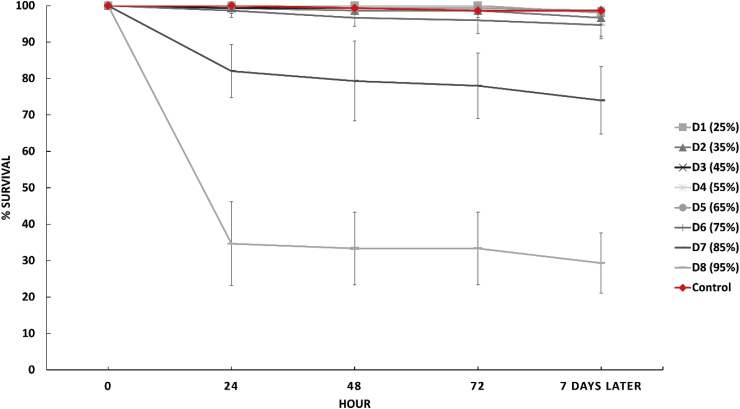
Average larval survival percentage on Control and D1 to D8 (waragi content) with SD.

Fig. 1 shows the average larval survival percent over the initial 72 h of the trial and at the end of the trial, when prepupae were observed, which occurred 10 days after the start of the experiment.

Of the eight dosage levels only two (D7 and D8) showed a significant effect on larval survival (*p* < .001). While D7 reduced survival by approximately 25% over the course of the trial, LC_50_ was only seen at D8, 95% inclusion of waragi waste. Within 24 h of exposure D8 had killed nearly 65% of the larvae, while D7 had killed less than 20% and survival stayed well over 95% for all other dosage groups. This result must be considered in light of the fact that the D8 diet was 95% waragi waste. Given the liquid state of the waragi waste and thus the high moisture content of the D8 and D7 diets, drowning cannot be excluded as the potential cause of death rather than toxicity from the waragi waste itself. Previous research has demonstrated the *H. illucens* eggs do not mature in feed with a moisture content above 80%, and similar observations of failure to thrive have been seen in larvae kept in high moisture feeds (Cheng et al., 2017; Fatchurochim et al., 1989). Further to this, waragi waste likely lacks sufficient nutrients to support larval development.

Studies have established that in addition to sufficient moisture in feed, protein and fat content are the next two largest determinants of larvae thriving (Oonincx et al., 2015a, Oonincx et al., 2015b). Thus, the lack of these two components may be the reason larvae fed on higher concentrations of waragi waste failed to thrive or died in greater numbers.

**Fig. 2:**
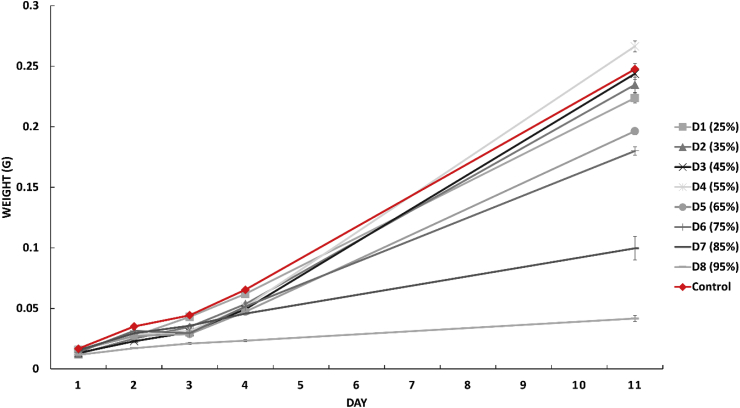
Average individual larval weight and growth rate on Control and D1 to D8 (waragi content) with SE.

This failure to thrive is also seen in the results of the individual larval growth rates in Fig. 2. Overall, a significant effect of dosage on end larval weight and average daily gain (*p* < .001) was seen.

Larvae on D5, D6, D7 and D8 had significantly lower end weights (*p* < .001) than the larvae on the control diet. D1, D2, and D3 all had significantly higher end weights than D4 to D8 (*p* < .001). The initial weight gain was also lower in all of the dosages when compared with the control, although some of the dosage groups (D1, D2, D3 & D4) did catch up and attained similar or greater end weights than the control group, though this difference was not significant. D7 and D8 displayed particularly low growth, with D7 larvae attaining only at most half of the final weight of the control group and D8 larvae weighing 5 times less than the control. In addition to this, D7 and D8 larvae showed no signs of pupation at the end of the trial, while all other dosages did.

There are two additional trends within the results which lend support to the waragi waste playing at least some part in the larvaes' failure to thrive and death, even if it is not entirely responsible. Larvae in the D6 group showed significantly decreased survival rates and impaired individual growth (*p* < .001). However, the moisture content (75%) of the D6 diet was well within established limits for successfully rearing larvae (Cheng et al., 2017; Diener et al., 2009). This was seen again when examining the individual weights. D5 preformed significantly (*p* < .001) worse than the lower dose groups and the control although they have an equivalent moisture content. This difference in performance is likely due to the increased exposure to and consumption of the waragi waste, either due to toxicity or nutritional deficiency suggesting that the low survival in D7 and D8 may not be entirely accounted for by drowning. This is further supported by the death trend seen in D7 and D8. There is an initial steep drop off in survival for both D7 and D8 and then it levels out again, with no further significant death in the groups. If the death was entirely caused by drowning it would be expected that the entire group would ultimately die, however as this was not the case some portion of the death is presumably down to exposure to the waragi waste and some larvae may be more resistant to the toxic aspects of it than others. Future trials should establish definitively if the waragi waste is the cause by drying it and incorporating it into the diet on a dry basis thus allowing the moisture content to be held consistent across dosages. However, care would need to be taken to ensure that no properties of the waragi waste which may contribute to toxicity are lost in this process.

In insect production both survival and end weight are critical parameters for determining success of a feed ingredient. As such, although larvae can survive on high doses of waragi waste within their diet, they only grow at equivalent or better rates to the control on diets which include ≤55% waragi waste.

### 3.2 Feeding trial

Table 3 displays the average end larval size, the calculated average daily weight gain per larvae, the percent of larvae which survived to pupation and the percent of pupae which successfully emerged as adults.

**Table 3:** Average end larval size, daily weight gain, colony survival percentage, and adult emergence percentage (±SD). If superscripts in the same column have no letters in common, means they differ significantly (Scheffé’s posthoc test; *p* < .05).

Diet	End larval size (g)	Average daily weight gain (g)	Survival %	Adult Emergence %
N (per diet)^a^	45	45	3	3
Control	0.24 (0.03)^b^	0.025 (0.003)^b^	98.56 (1.02)	98.32 (1.47)
CP	0.26 (0.04)^a^	0.029 (0.005)^a^	99.44 (0.51)	97.54 (1.03)
GN	0.24 (0.03)^a^	0.027 (0.005)^a^	98.67 (0.67)	95.72 (5.08)
SSM	0.23 (0.03)^b^	0.025 (0.003)^b^	98.56 (1.39)	87.62 (10.40)
CSC	0.25 (0.04)^a^	0.028 (0.004)^a^	99.78 (0.38)	95.33 (2.30)

a N refers to the number of individual larvae in the case of end larval size and average daily weight gain, and the number of biological replicates in the case of survival and adult emergence.

There was no significant effect of diet on survival percent. Larvae on the CP diet and CSC diet had the highest survival, though only by approximately 1%. However, there was a significant effect of diet on the end larval size and average daily weight gain (*p* < .001). Larvae fed on the CP diet were, on average, larger at the end of the trial and gained more weight daily, though they were only significantly (*p* < .001) larger than larvae on the control or SSM diet. This difference is potentially due to that fact that they received the highest quality protein, chicken offal, of all the groups. Research has previously shown that larvae perform exceedingly well on animal-based protein sources (St-Hilaire et al., 2007).

There was also no significant effect of diet on adult emergence rates. The CP and Control diets showed the highest adult fly emergence rates, achieving close to 100% emergence. On the SSM diet, 10% fewer adult flies emerged than all other diets. Diet is known to play a role in larval and adult survival and development, significant changes have previously been seen in diets which were not nutritionally adequate (Gobbi et al., 2013). However, these differences have generally been linked to survival and growth rather than adult emergence rates. As survival and growth were not impaired in the SSM group it is not clear at this stage what may have impacted the adult emergence rates.

All diets had a larval development time of between 18 and 22 days, which is quicker than has been reported for some previous research utilising other waste sources where development times have been reported to take over 100 days at times (Oonincx et al., 2015a, Oonincx et al., 2015b). This difference in development times is most likely accounted for by the age of the larvae at the start of the trial. In this study larvae were used after first being reared for seven days on a starter diet, as has been standard in other research to facilitate accurate counting and weighing (Li et al., 2011; Myers et al., 2008). Trials which report quick development rates (<25 days) generally only introduced larvae to the waste-based diet after several days on a ‘starter’ diet, as was done in this trial (Meneguz et al., 2018; Rehman et al., 2017; Diener et al., 2009). In trials were larvae were directly introduced to waste feed from hatching considerably more variation is seen in development time and overall survival (Alyokhin et al., 2018). It is suggested that larval age at transfer to a waste source and the diet quality in early development play a role in the subsequent development time of the larvae (Oonincx et al., 2015a, Oonincx et al., 2015b). As such future work should determine if the above diets are subtle to be used from hatching with similar positive results or if a specialisted starter diet is still needed for the larvae.

**Fig. 3:**
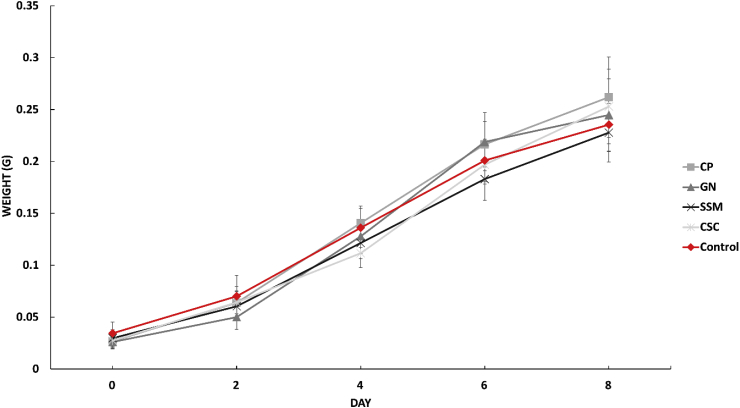
Average individual larval growth over time with SD.

Although these differences exist it is evident that the inclusion of 25% waragi did not impact the overall survival and development of the larvae, but rather the individual feed compositions likely account for variations in growth and weight. The results on individual larvae growth in Fig. 3 support this.

Two of the four trial diets, CP and GN, out preformed the control diet at Day 6, with individual larvae weighing on average 0.01 g more at this point, although only the CP and CSC fed larvae had a significantly (*p < .01)* larger average daily weight gain. This growth difference does even out as larvae move closer to pupation but is a notable difference if the aim is to produce a certain weight of larvae quickly. All larvae showed signs of pupation within 24 h of each other, indicating the diets and specifically the presence of the waragi waste did not impede development.

## 4 Conclusions

The dietary toxicity trial provides clear indications that *H. illucens* larvae can readily survive on waragi waste at an inclusion percentage up to 85% without significant impact on survival or growth. It is also plausible that at higher inclusion percentages (>75%) larval death is due to drowning or nutrient deficiency rather than toxicity, although individual growth data does suggest at least some stunting caused by the inclusion of waragi waste at high percentages. The further feeding trial with a 25% inclusion of waragi waste in the diet demonstrated no differences in survival between trial and control diets, however there was an effect on end weight and average daily gain; however, this is likely explained by the slight differences in feed composition rather than due to the waragi as inclusion was kept constant across diets.

Overall, the data demonstrates that the larve of *H. illucens* can successfully be used to process waragi waste in combination with other locally available feed stuffs or wastes; as demonstrated by survival rates higher than reported in previous research of larvae on waste sources (Gobbi et al., 2013; Myers et al., 2008; Oonincx et al., 2015a, Oonincx et al., 2015b). This finding presents a unique opportunity for communities in Uganda to make use of an otherwise useless substance.

It is paramount to note that these finding should not encourage increased production of waragi waste to feed larvae. Although the larvae can process the waragi waste it is not essential that they do; they are able to thrive equally well on diets without waragi waste. Feeding the waragi waste to the larvae should only be used as a short-term environmental protection mechanism, not a long-term breeding protocol, while solutions can be found to reduce waragi brewing. One such solution may be to divert molasses from waragi brewing to *H. illucens* rearing to then sell on into the animal feed system, providing the income that the waragi brewing otherwise would.

Thus, future research should focus strongly on strategies to combat illegal waragi brewing first and foremost, while also identifying methods to provide stable income for communities. Rearing *H. illucens* larvae as animal feed may be one solution to providing income. Continued research should endeavour to develop production systems most appropriate in situ and to continue to refine the ideal feed source for rearing larvae in an economically and environmentally sustainable way. Research should also ensure that the waragi waste composed diet does not impact the overall safety of the larvae as an animal feed.

## References

[bib1] Alyokhin A., Buzza A., Beaulieu J. (2018). Effects of food substrates and moxidectin on development of black soldier fly, *Hermetia illucens*. J. Appl. Entomol..

[bib2] Banks I.J., Gibson W.T., Cameron M.M. (2014). Growth rates of black soldier fly larvae fed on fresh human faeces and their implication for improving sanitation. Trop. Med. Int. Health.

[bib3] Barragan-Fonseca K.B., Dicke M., van Loon J.J.A. (2017). Nutritional value of the black soldier fly (*Hermetia illucens* L.) and its suitability as animal feed: a review. J. Insects Food Feed..

[bib4] Bondari K., Sheppard D. (1987). Soldier fly, *Hermetia illucens* L., larvae as feed for channel catfish, Ictalurus punctatus (Rafinesque), and blue tilapia, Oreochromis aureus (Steindachner). Aquacult. Res..

[bib5] Cheng J.Y.K., Chiu S.L.H., Lo I.M.C. (2017). Effects of moisture content of food waste on residue separation, larval growth and larval survival in black soldier fly bioconversion. Waste Manag..

[bib6] Copello A. (1926). Biologia de *Hermetia illucens* Latr. Rev. Soc. Entomol. Argentina..

[bib7] Diener S., Zurbrügg C., Tockner K. (2009). Conversion of organic material by black soldier fly larvae: establishing optimal feeding rates. Waste Manag. Res..

[bib8] Elunya J. (2009). River Namatala Polluted by Waragi Distillers: Uganda Radio Network.

[bib9] Elwert C., Knips I., Katz P. (2010). A novel protein source: maggot meal of the black soldier fly (Hermetia illucens) in broiler feed. Tagung Schweine-und Geflügelernährung.

[bib10] Erickson M., Islam M., Sheppard C. (2004). Reduction *of Escherichia coli* O157: H7 and *Salmonella enterica serovar enteritidis* in chicken manure by larvae of the black soldier fly. J. food..

[bib11] Fatchurochim S., Geden C.J., Axtell R.C. (1989). Filth fly (Diptera) oviposition and larval development in poulty manure of various moisture levels. J. Entomol. Sci..

[bib12] Furman D., Young R., Catts P. (1959). *Hermetia illucens* (Linnaeus) as a factor in the natural control of Musca domestica Linnaeus. J. Econ..

[bib13] Gatsiounis I. (2010). The Battle to Stop Drink from Destroying Uganda - TIME. Time.

[bib14] Gobbi P., Martínez-Sánchez A., Rojo S. (2013). The effects of larval diet on adult life-history traits of the black soldier fly, *Hermetia illucens* (Diptera: Stratiomyidae). Eur. J. Entomol..

[bib15] Hale O. (1973). Dried *Hermetia illucens* larvae (Diptera: Stratiomyidae) as a feed additive for poultry. Ga Entomol Soc J.

[bib16] Heath A. (2015). Uganda's Ongoing Struggle with Moonshine.

[bib17] Kasamba C., Mugo K. (2007). A Ugandan River under Siege.

[bib18] Lalander C., Diener S., Magri M., Zurbrügg C. (2013). Faecal sludge management with the larvae of the black soldier fly (*Hermetia illucens*)—from a hygiene aspect. Sci. Total..

[bib19] Li Q., Zheng L., Qiu N., Cai H., Tomberlin J.K., Yu Z. (2011). Bioconversion of dairy manure by black soldier fly (Diptera: Stratiomyidae) for biodiesel and sugar production. Waste Manag..

[bib20] Liu Q., Tomberlin J., Brady J., Sanford M. (2008). Black soldier fly (Diptera: Stratiomyidae) larvae reduce *Escherichia coli* in dairy manure. Environ. Entomol..

[bib21] McDermott J., Staal S., Freeman H., Herrero M. (2010). Sustaining intensification of smallholder livestock systems in the tropics. Livest. Sci..

[bib22] Meneguz M., Schiavone A., Gai F., Dama A., Lussiana C., Renna M., Gasco L. (2018). Effect of rearing substrate on growth performance, waste reduction efficiency and chemical composition of black soldier fly (*Hermetia illucens* ) larvae. J. Sci. Food Agric..

[bib23] Myers H.M., Tomberlin J.K., Lambert B.D., Kattes D. (2008). Development of black soldier fly (Diptera: Stratiomyidae) larvae fed dairy manure. Environ. Entomol..

[bib24] Newton L., Sheppard C., Watson D. (2005). Using the black soldier fly, *Hermetia illucens*, as a value-added tool for the management of swine manure. Anim. Poult..

[bib25] OECD (2012). Bumblebee, acute oral toxicity test 247. Guidel. Test. Chem..

[bib26] OECD (1995). Honey Bee (Apis mellifera L.), Chronic Oral Toxicity Test 245 (10-Day Feeding), Guidelines for the Testing of Chemicals.

[bib27] OECD (1984). Avian Dietary Toxicity Test 205, Guidelines for the Testing of Chemicals.

[bib28] Oonincx D.G., Van Broekhoven S., Van Huis A., Van Loon J.J. (2015). Feed conversion, survival and development, and composition of four insect species on diets composed of food by-products. PloS One.

[bib29] Oonincx D.G., van Huis A., van Loon J.J. (2015). Nutrient utilisation by black soldier flies fed with chicken, pig, or cow manure. J. Insects Food Feed..

[bib30] Prüss-Üstün A., Bos R., Gore F., Bartram J. (2008). Safer water, better health. World Health.

[bib31] Rehman K. ur, Rehman A., Cai M., Zheng L., Xiao X., Somroo A.A., Wang H., Li W., Yu Z., Zhang J. (2017). Conversion of mixtures of dairy manure and soybean curd residue by black soldier fly larvae (*Hermetia illucens* L.). J. Clean. Prod..

[bib32] Republic of Uganda, n.d. A Study on Sentencing and Offences Legislation in Uganda Part A: Summary and Recommendations.

[bib33] Sealey W.M., Gaylord T.G., Barrows F.T., Tomberlin J.K., McGuire M.A., Ross C., St-Hilaire S. (2011). Sensory analysis of rainbow trout, *Oncorhynchus mykiss*, fed enriched black soldier fly prepupae, *Hermetia illucens*. J. World Aquacult. Soc..

[bib34] Sheppard C. (1983). House fly and lesser fly control utilizing the black soldier fly in manure management systems for caged laying hens. Environ. Entomol..

[bib35] Sheppard D., Newton G., Thompson S. (1994). A value added manure management system using the black soldier fly. Bioresour. Technol..

[bib36] Ssebuyira M. (2014). Water Quality Has Reduced - Nema - Daily Monitor.

[bib37] Ssekika E. (2015). Uganda: Disaster Looms as Masindi Factory Pollutes River - allAfrica.Com. Obs.

[bib38] St-Hilaire S., Cranfill K., McGuire M.A., Mosley E.E., Tomberlin J.K., Newton L., Sealey W., Sheppard C., Irving S. (2007). Fish offal recycling by the black soldier fly produces a foodstuff high in omega-3 fatty acids. J. World.

[bib39] Tomberlin J.K., Adler P.H., Myers H.M. (2009). Development of the black soldier fly (Diptera: Stratiomyidae) in relation to temperature. Environ. Entomol..

[bib40] Tschirner M., Simon A. (2015). Influence of different growing substrates and processing on the nutrient composition of black soldier fly larvae destined for animal feed. J. Insects Food..

[bib41] Tumwine J., Munguti K. (2002). 30 Years of Change in Domestic Water Use & Environmental Health in East Africa: Uganda Country Study.

[bib42] UNICEF, WHO (2017). Progress on Drinking Water.

[bib43] USDA, n.d. Food composition databases show foods -- millet flour [WWW Document]. URL https://ndb.nal.usda.gov/ndb/foods/show/6642?fgcd=&manu=&lfacet=&format=&count=&max=50&offset=&sort=default&order=asc&qlookup=millet&ds=&qt=&qp=&qa=&qn=&q=&ing= (accessed 7.18.2017).

